# MPAR-RCNN: a multi-task network for multiple person detection with attribute recognition

**DOI:** 10.3389/frai.2025.1454488

**Published:** 2025-02-07

**Authors:** S. Raghavendra, S. K. Abhilash, Venu Madhav Nookala, Jayashree Shetty, Praveen Gurunath Bharathi

**Affiliations:** ^1^Department of Information and Communication Technology, Manipal Institute of Technology, Manipal Academy of Higher Education, Manipal, India; ^2^KPIT Technologies, Bengaluru, India; ^3^Nuclear Medicine and Molecular Imaging, Department of Radiology, Stanford Medicine, Palo Alto, CA, United States

**Keywords:** attribute recognition, convolution neural network, human attribute recognition, multi-task learning, object detection

## Abstract

Multi-label attribute recognition is a critical task in computer vision, with applications ranging across diverse fields. This problem often involves detecting objects with multiple attributes, necessitating sophisticated models capable of both high-level differentiation and fine-grained feature extraction. The integration of object detection and attribute recognition typically relies on approaches such as dual-stage networks, where accurate predictions depend on advanced feature extraction techniques, such as Region of Interest (RoI) pooling. To meet these demands, an efficient method that achieves both reliable detection and attribute classification in a unified framework is essential. This study introduces an innovative MTL framework designed to incorporate Multi-Person Attribute Recognition (MPAR) within a single-model architecture. Named MPAR-RCNN, this framework unifies object detection and attribute recognition tasks through a spatially aware, shared backbone, facilitating efficient and accurate multi-label prediction. Unlike the traditional Fast Region-based Convolutional Neural Network (R-CNN), which separately manages person detection and attribute classification with a dual-stage network, the MPAR-RCNN architecture optimizes both tasks within a single structure. Validated on the WIDER (Web Image Dataset for Event Recognition) dataset, the proposed model demonstrates an improvement over current state-of-the-art (SOTA) architectures, showcasing its potential in advancing multi-label attribute recognition.

## 1 Introduction

The task of human analysis is fundamental for real-time understanding of individuals in various scenarios. It demands highly detailed human features at the pixel level and involves several cognitive processes, such as detection, segmentation, and estimation (Poulose et al., 2022). In real-time deployment of models, balancing both accuracy and speed is essential for effective algorithm performance. A model's ability to function in real-time environments depends heavily on these two factors as they create a critical ecosystem for its applications. The advancements in convolutional neural networks (CNNs) have made the extraction of human or object features from images not only faster but also more precise, facilitating more sophisticated real-time analysis (Edriss et al., 2024). Recently, CNN-based object detection algorithms have evolved into two main approaches. The first is the two-stage approach, where a regional proposal network is integrated with a CNN, as seen in the R-CNN model proposed by Girshick et al. (2014). This approach first identifies potential regions of interest and then uses CNNs to further analyze those areas. In contrast, the second approach is the single-stage method, which simplifies object detection into a regression problem. Models such as You Only Look Once (YOLO), as introduced by Bochkovskiy et al. (2020), detect objects in a single pass, offering improved speed for real-time applications.

The introduction of R-CNN marked a pivotal advancement, leveraging a large CNN to examine suggested areas, which influenced the development of subsequent multi-stage algorithms. The R-CNN's structure and capabilities contributed to making CNN-based object detection a mainstream approach. This breakthrough enabled models to achieve high precision while also adapting to the fast-paced needs of real-time applications, significantly advancing the field of object detection. In 2015, Fast R-CNN, developed by Girshick (2015), built on the original R-CNN architecture by separating the fully connected layer's output into two distinct vectors: one for bounding box (BBox) regression and another for classification scores using softmax. This approach introduced the region of interest (RoI) pooling layer, which was based on the spatial pyramid pooling (SPP) layer from SPP-Net. The incorporation of RoI pooling solved challenges related to both object classification and bounding box regression, marking a significant advancement in object detection. In addition, Fast R-CNN optimizes storage by saving interpretations and detections in cache memory and replacing singular value decomposition (SVD) with softmax, thereby reducing storage space and enhancing processing speeds. Following Fast R-CNN, dual-stage networks such as Mask R-CNN (He et al., 2017) were developed to improve human detection by predicting both boundary boxes and class-aware mask predictions in parallel using extensive convolutional layers. This evolution paved the way for MTL techniques, as explored by Zhu and Wu (2021) which leverage shared representations across tasks to address both time and memory constraints in real-world applications. This single-model approach enables highly efficient and accurate real-time detection in dynamic environments, making it especially valuable for applications requiring rapid processing.

The motivation to advance human analysis lies in the need for accurate, real-time understanding of individuals in various dynamic scenarios. This requires pixel-level precision for tasks such as detection, segmentation, and estimation, especially in challenging conditions such as low lighting and overlapping objects. Effective algorithms must balance high accuracy with processing speed to support real-time deployment. Convolutional neural networks (CNNs) have significantly improved human analysis by enhancing both speed and precision. Innovations such as Fast R-CNN and multi-task learning (MTL) have further refined these methods, making sophisticated human analysis feasible in demanding real-world settings. MPAR-RCNN framework enhances multi-person detection by visualizing bounding boxes and attribute details. The framework integrates a region proposal network (RPN) for efficient image parsing and region prediction. Unlike traditional methods such as selective search, RPN utilizes neural networks to generate proposals directly from feature maps, significantly accelerating detection. By optimizing these region proposals, MPAR-RCNN delivers faster and more precise human detection, making it a powerful tool for applications in various complex scenarios. The contributions of the study are listed below:

1. A novel MPAR-RCNN framework is introduced, which subdues the challenges of gradient loss for previous MTL frameworks.
2. An effective MTL pipeline is implemented with a single shared backbone and experimented with various backbones being cognizant of the number of parameters and their mAP levels.
3. Additional ablation studies were performed with different augmentation techniques and other changes in hyperparameters with different datasets to illustrate the flexibility of MPAR-RCNN.

The layout of the study encompasses as follows: Section 2 portraits the details of the related works, and the proposed methodology is elaborated in Section 3. Section 4 contains the experimental settings of the proposed Model and its datasets and performance metrics used. The results and their discussion is given in Section 5. Section 6 contains the details of the ablation studies, and Section 7 mentions the conclusion and intended future works.

## 2 Related works

In this section, the different methods of OD, along with existing human attribute recognition architectures, are discussed. The state of art models is mentioned and compared with the desideratum of MTL frameworks.

### 2.1 Single stage v/s multi-stage object detection

OD is a process that involves identifying and categorizing objects in an image, and labeling them with a rectangular bounding box (BBox) to indicate their location. There are two main categories of OD frameworks. The first category generates region proposals and then classifies each proposal into different object types. The second category views OD as a classification or regression problem, using a unified framework to determine the category and position of an object.

The majority of the region proposal-based approaches, some of which are correlated with one another, include R-CNN (Gkioxari et al., 2015), Faster R-CNN (Ren et al., 2015), and Mask R-CNN (He et al., 2017). Regression or classification-based methods include MultiBox (Liu et al., 2016), G-CNN (Song et al., 2019), Single Shot multibox Detector (SSD) (Liu et al., 2016), and Yolov4 (Bochkovskiy et al., 2020). Faster RCNN uses anchors to bridge the gap between these two approaches. One-stage object detectors are faster but struggle with small objects or objects with unusual shapes (Liu et al., 2016). The MPAR-RCNN framework uses a detection head with different architecture to improve the classification and detection of objects using region-based methods. Fast R-CNN is used to extract image features, and the RoI pooling layer is used to feed the fully connected layer. MPAR-RCNN framework performs OD and attribute recognition together with fewer parameters compared to single stage OD and attribute recognition. Table 1 provides a comparison of the differences between MPAR-RCNN and other frameworks. Overall, effective OD frameworks are essential for a wide range of applications, from self-driving cars to facial recognition technology.

**Table 1:** High-level comparison of OD with attribute recognition (AR) and multi-task learning (MTL) frameworks using standard datasets.

**Category**	**Architecture**	**Backbone**	**Function**	**Params**	**MACs**	**Dataset**	**BBox**	**Attributes**
				**(M)**	**(G)**		**(AP)**	**(mA)**
Attribute recognition	R*-CNN (Gkioxari et al., 2015)	16-Layer CNN	AR			WIDER-Attribute		80.5
	DHC (Li et al., 2016)	16-Layer CNN		-	-			81.3
	VeSPA (Sarfraz et al., 2017)	Inception v1		17	3.5			82.4
	ResNet-101+MTL+CRL (Dong et al., 2017)	ResNet-101		60	127			84.7
	ResNet-SRN (Dong et al., 2017)	ResNet-107		-	-			86.2
	SEA (Sarafianos et al., 2018)	ResNet-101		45	8			86.4
	Inception-V3 (Szegedy et al., 2016)	Inception v3		24	6			85.86
	SA (Kalayeh and Shah, 2019)	Inception v3		24	6			87.58
Multi task learning	HEIR-RCNN (Yang et al., 2020a)	ResNet50-FPN	OD + HPD	36	215.88	COCO Human Parts	36.8	
	RP-RCNN (Yang et al., 2020b)	ResNet50-GSE-FPN	OD + HPS	57.59	740.53	COCO	44.1	
	Multitask CenterNet (Heuer et al., 2021)	Hourglass-104	OD + FLD + KPD	60.81	-	COCO	8.42	
	MPAR-RCNN - Small	MogaNet	OD + AR	8.42	52.6	WIDER	56.5	91.73
	MPAR-RCNN - Medium	MogaNet	OD + AR	14.8	64.8	WIDER	60.06	92.27
	MPAR-RCNN - Large	MogaNet	OD + AR	26	168.35	WIDER	**61.4**	**93.98**

COCO and WIDER-Attribute datasets are commonly used for evaluating different architectures with their different functions like OD with object segmentation (OD + OS). Other functions include human part segmentation (HPS), human part detection (HPD), and key point estimation (KPD). Bold values indicate metrics obtained using this approach.

### 2.2 Human attribute parsing

It has become common to describe human beings using a set of characteristics known as attributes. Human attribute parsing techniques focus on identifying body parts or sections that are closely connected to the features and calculate attributes associated with them to distinguish their properties. The human attribute parsing method is useful for tasks such as person re-identification, person retrieval, person detection, and attribute-based representations. However, due to various vantage points or lighting situations, the visual appearances of the same person may differ noticeably across many perspectives. To overcome this, human attribute parsing methods first identify high-level characteristics that remain the same for most of the time frames in all scenarios for the same people, such as their clothing, and then use them to match the identities of samples. It has been established that people's characteristics directly affect detection abilities. Some techniques use body language, either through body poses or by focusing on the torso, leg, or head areas, to predict future behavior. Others analyze the appearances of both people and their surroundings to better understand the scenarios.

Human semantic parsing (Moghaddam et al., 2021) is a technique that helps locate different attributes of a human body by extracting the contours of various parts of the body. To do this, a pre-trained parsing model is used that interprets each pixel of the image. Xia et al. (2017) proposed a fully connected CRF framework that incorporates human instance clustering and joint labeling. In this framework, an approximated pose information is used by a fully connected layer for further processing. However, due to the time and cost it takes to produce ground truth data, there may be a lot of label noise. Mordan et al. (2021) have also studied joint pedestrian detection and attribute recognition, which is similar to the suggested approach.

Performing detection using a multi-stage multi-head approach makes the planned MTL network forward pass time-consuming. They also integrate attributes in a multi-stage manner, but MPAR-RCNN advances this strategy by learning around these attributes and adding a normalization in backpropagation to address gradient scale problems during training. The proposed framework uses the WIDER-Attribute (Jia et al., 2024) dataset, which has 14 binary attributes.

### 2.3 Multi-task learning frameworks

MTL (Crawshaw, 2020) is a technique that aims to optimize the distribution of the learning capacity across multiple tasks to improve their performance and minimize the computational demands. However, recent studies (Kokkinos, 2017) have shown that the benefits of MTL are not always consistent and depend on the specific objectives of the tasks. To achieve an optimal transfer between tasks, the system must be customized to the particular target objectives. MTL is effective in improving autonomous vehicle resilience to adversarial attacks (Rasouli and Tsotsos, 2019), a critical safety issue. There are two primary approaches to implementing MTL. Some methods aim to enhance the network structures (Misra et al., 2016) or increase the degree of task sharing. On the other hand, leverage the learning loss weights of any given architecture to balance tasks (Chen et al., 2018), or manipulate gradients to minimize negative transfer. In the MPAR-RCNN framework, MTL has been successfully implemented using the logit gradients from the convolution layers to reduce the computations with minimal accuracy loss while using dual-stage networks.

## 3 Proposed methodology

**Figure 1:**
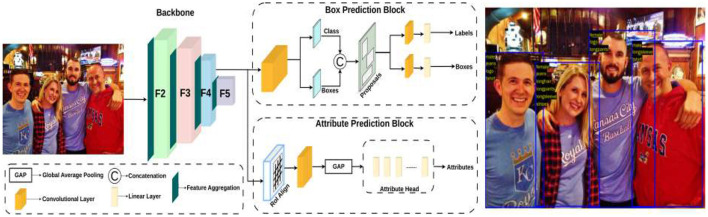
Architecture pipeline of MPAR-RCNN framework with MTL. It contains the regional proposed network with a detection and attributes head for recognition. Reproduced with permission from ‘Recognize Complex Events From Static Images by Fusing Deep Channels' by Xiong et al via Web Image Dataset for Event Recognition [WIDER], https://paperswithcode.com/dataset/wider, licensed under CC BY-SA 4.0.

The architecture of multi-person attribute recognition region convolutional neural networks (MPAR-RCNN) is illustrated in Figure 1. In this section, we will explain each functionality of this architecture, which is used for detecting multiple persons with various attributes in a multi-task environment.

### 3.1 Backbone

The input sequence images are first fed into the embedding stem to regulate the feature resolutions. Assuming the input image in H × W resolutions, features of the four stages are in 4 × 4, 8 × 8, 16 × 16, and 32 × 32, resolution respectively. Then, the embedded feature flows into multi-stage gated aggregation consisting of two branches: the aggregation branch and the context branch. The aggregation branch is responsible for generating gate weights. The context branch performs multi-scale feature extraction through convolution with different kernel sizes and different hole sizes, thereby capturing multi-order interactions of context. It is worth noting that the outputs of the two branches use the SiLU activation function (SILU has both Sigmoid gating effect and stable training characteristics).

### 3.2 Box prediction block

The fully convolutional network known as the region proposal network (RPN) predicts object limits and objectness scores at each place simultaneously. The RPN is fully trained to produce excellent region suggestions that indicate where to search and where the object is located. With a broad range of scales and aspect ratios, region suggestions can be predicted with efficiency using RPNs. It makes use of anchor boxes as references at various aspect ratios and scales. Regression references can be envisioned as a pyramid-shaped method that circumvents the need to count images or filters with different aspect ratios or sizes. R-CNN extracts these boxes–known as regions–from an image by using selective search. First, the R-CNN labels the bounding boxes and class labels of the extracted region proposals (e.g., anchor boxes can also be considered as region proposals) from the input image. Next, each region proposal is subjected to forward propagation using CNN to extract its features. The class and bounding box of each region proposal are then predicted using the attributes of that region proposal.

### 3.3 Attribute prediction block

RoIAlign is an operation for extracting a small feature map from each RoI in detection and segmentation-based tasks. It removes the harsh quantization of RoI Pool, properly aligning the extracted features with the input. To avoid any quantization of the RoI boundaries, RoIAlign uses bilinear interpolation to compute the exact values of the input features at four regularly sampled locations in each RoI boundary, and the result is then aggregated (using max or average). RoIAlign is used for parsing the ROI-based features for objects with the help of C4 convolution layer. The features are upscaled and given to the convolution layer succeeded by the global average pooling layer to extract the logit bits for the attribute head. These features are then passed to the fully convolutional (FC) layers to get the probability values to produce the accurate attributes after a certain threshold value. The WIDER-Attribute dataset is used for training in the proposed model. This dataset contains 14 binary person attributes some of which include gender, clothes, facemasks, and other accessories. In the MTL pipeline, the detection and attributes are jointly used and the computation values of gradients are highly reduced while getting over a percentage increase compared to the SOTA models. A *n*×*n* spatial window of the input convolutional feature map serves as the input for this tiny network.

### 3.4 Loss function

The MPAR-RCNN is an MTL pipeline with a dual-stage network which indicates that the overall single loss function of the framework is the sparse softmax cross entropy. The loss at different stages of the MPAR-RCNN framework can be summarized as regression loss, bounding box loss, and attribute loss.

#### 3.4.1 Regression loss

The loss from the detection head can be segmented into the Regression Loss *L*_*reg*_ and the Huber loss *L*_*huber*_. The regression loss is formulated as follows:


(1) $$\begin{array}{l} L_{reg}=\overset{D}{\underset{i=1}{\sum }}|y_{i}-x_{i}| \end{array}$$

where *x* and *y* are *D* dimensional vectors, and *y*_*i*_ denotes the value on the *i*th dimension of *y*.

#### 3.4.2 Huber loss

One often used metric to quantify the discrepancy between expected and actual results in regression situations is the Huber loss. The average squared difference between the actual and projected values is what this metric measures.


(2) $$L_{huber}=\{\begin{array}{cc} \frac{1}{2}{\left(y-f(x)\right)}^{2} & for\left|\left(y-f(x\right)\right|\leq \delta \\ \delta \left|\left(y-f(x\right)\right|-\frac{1}{2}\delta ) & otherwise \end{array}$$

where δ is the number of BBoxes from the detection head, and *f*(*x*) and *y* are the predicted box and the ground truth (GT) label, respectively.

#### 3.4.3 SoftMax cross entropy loss (attribute)

The loss function of the attribute head is the softmax cross entropy and is defined as follows:


(3) $$\sigma ({\vec{z}}_{i})=\frac{e^{z_{i}}}{\sum_{j=1}^{K}e^{z_{j}}}\;for\;i=1,2,\ldots ,K$$

where K is the number of attributes, and ${\vec{z}}_{i}$ is the *i*^*th*^ attribute label.

MPAR-RCNN framework considers regression loss, Huber loss, and attribute loss together to measure the overall performance of the model. The loss function of the MPAR-RCNN is formulated as the absolute summation of all the three loss functions as


(4) $$\begin{array}{l} L_{total}=L_{reg}+L_{huber}+L_{attr} \end{array}$$

## 4 Experiments

### 4.1 Datasets

**Figure 2:**
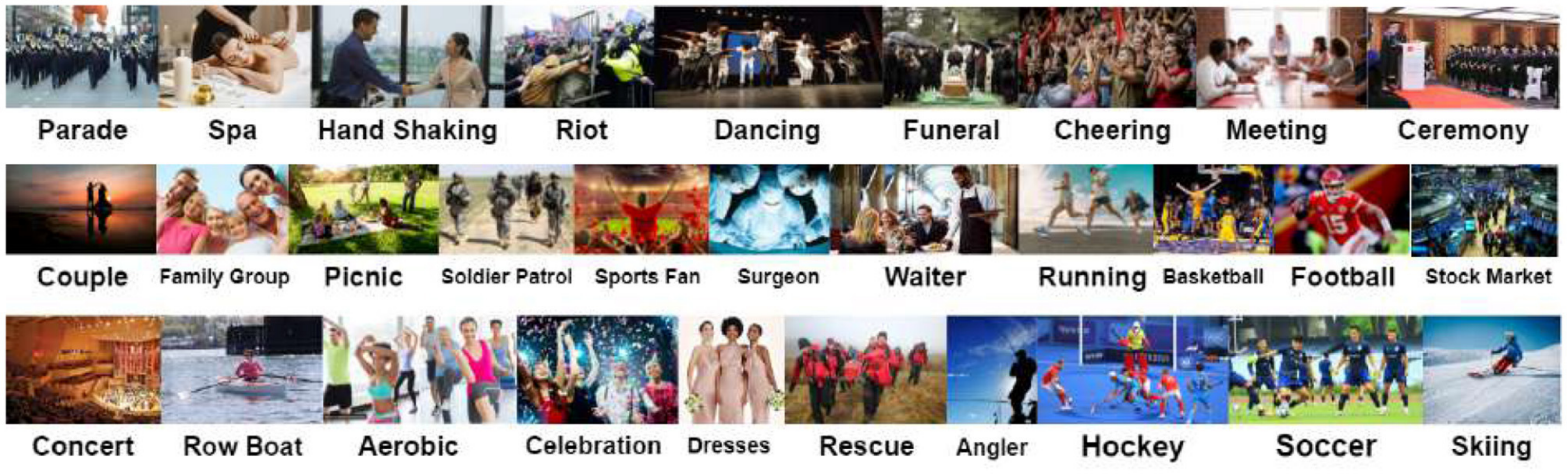
Sample images of WIDER-Attribute in different scenarios. Reproduced with permission from ‘Recognize Complex Events From Static Images by Fusing Deep Channels' by Xiong et al via Web Image Dataset for Event Recognition [WIDER], https://paperswithcode.com/dataset/wider, licensed under CC BY-SA 4.0.

**Figure 3:**
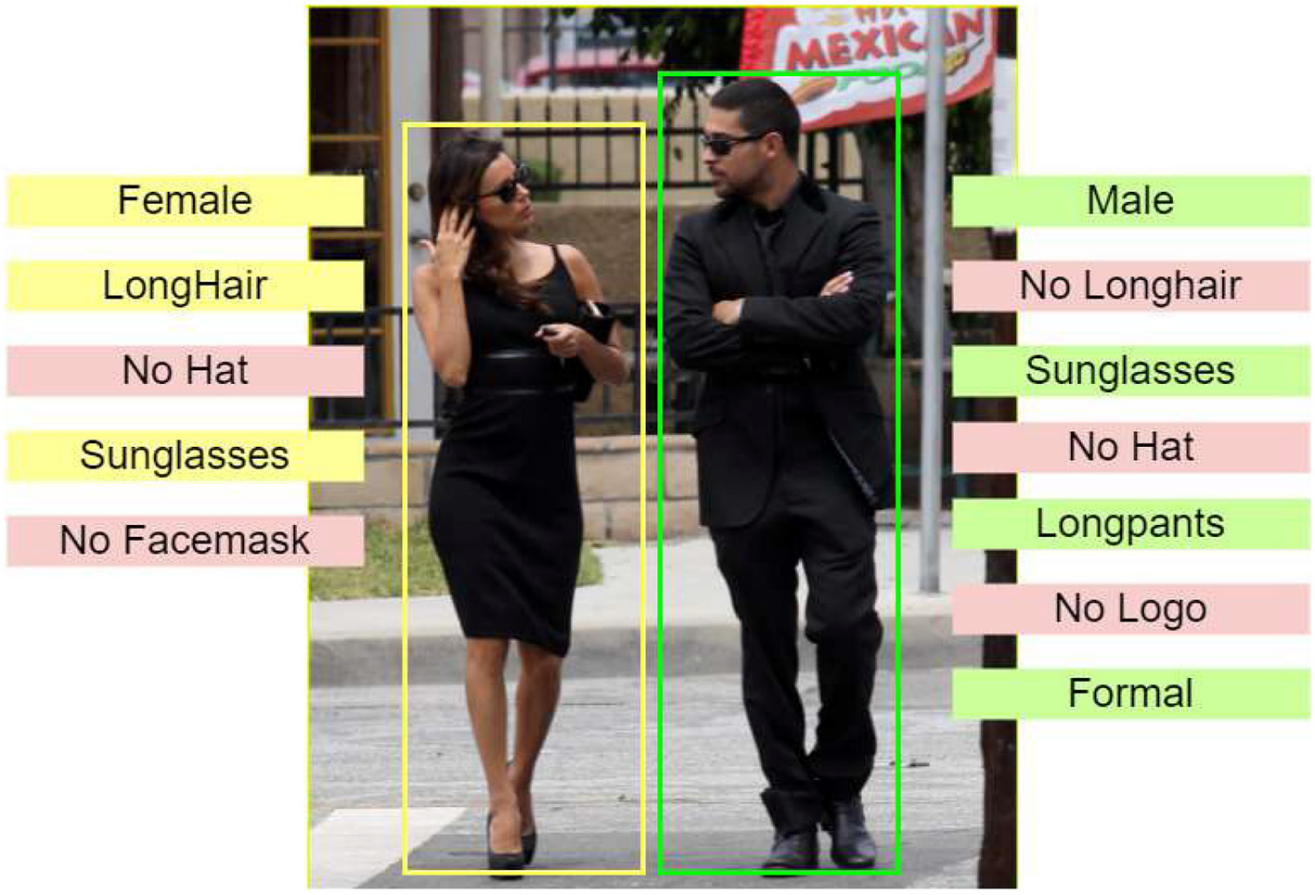
Sample image from WIDER-Attribute dataset with attribute recognition. Reproduced with permission from ‘Recognize Complex Events From Static Images by Fusing Deep Channels' by Xiong et al via Web Image Dataset for Event Recognition [WIDER], https://paperswithcode.com/dataset/wider, licensed under CC BY-SA 4.0.

WIDER-Attribute is a pedestrian dataset containing 14 different classes of categories to each person. The sample image from WIDER is shown in Figures 2, 3.

There are other attribute datasets such as JAAD, PARSE-27K, and Berkeley Attributes of People. The proposed framework is designed for detection with attributes, and it is evaluated and trained on the WIDER-Attribute dataset. This dataset has more explicit people and event descriptions. Researchers are able to study the advantages of modern person context thanks to the profusion of various visuals and human annotations. 28,345 annotated images are used for the training and validation sets whereas 29,179 individuals for the test set. The split is shown in Table 2. The training and validation set is used for training, and the test set is used to assess the performance in accordance with post-processing.

**Table 2:** Attribute-based dataset partitioning for training and testing in object detection.

**Attributes**	**Train**	**Validation**	**Test**
Male presenter	12,979	3,140	16,769
Longhair	5,148	1,212	6,428
Sunglass	1,179	244	1,378
Hat	5,235	1,172	6,994
Tshirt	4,994	1,217	5,820
Longsleeve	10,743	2,429	13,888
Formal	1,690	292	2,119
Shorts	3,385	985	4,278
Jeans	1941	372	2,354
Longpants	8,355	1,908	10,888
Skirt	2,340	510	2,884
Facemask	780	154	1,010
Logo	6,111	1,491	8,367
Stripe	1,207	331	1,808

### 4.2 Evaluation metrics

Mean average precision (mAP) and mean accuracy (mA) are the primary evaluation metric used for object detection, segmentation, and attribute recognition. The positive threshold is set as 0.5 for evaluating the average precision (AP). The mAP values are calculated over recall values from 0 to 1 based on the four metrics of the confusion matrix and intersection over union (IoU). Higher values of IoU indicate that the predicted BBox is close to the ground truth annotations. The precision value depends on the true positives of the correct predictions. For attributes, individual mA values are calculated and shown in Tables 1, 3.

**Table 3:** Evaluation of attribute-wise comparison of our proposed framework with 10 different methods and their backbones.

**WIDER-attribute**											
	**Fast-RCNN (Girshick**, **2015****)**	**R-CNN (Gkioxari et al.**, **2015****)**	**DHC (Li et al.**, **2016****)**	**ResNet101 (He et al.**, **2016****)**	**CAM (Guo et al.**, **2017****)**	**SEA (Sarafianos et al.**, **2018****)**	**Inception-V3 (Szegedy et al.**, **2016****)**	**SA (Kalayeh and Shah**, **2019****)**	**MPAR-RCNN**		
									**MPAR-RCNN - small**	**MPAR-RCNN - medium**	**MPAR-RCNN - large**
Male presenter	94	94	94	94	95	96	95.6	96.64	92.69	92.37	92.37
Longhair	81	82	82	85	85	88	86.98	89.25	86.11	91.15	91.15
Sunglass	60	62	64	69	71	74	70.56	78.31	95.36	95.29	96.29
Hat	91	91	92	91	94	93	92.87	95.04	91.01	90.61	90.61
tShirt	76	76	78	80	78	83	83.36	84.77	86.51	87.47	90.47
Longsleeve	94	95	95	96	96	96	96.71	97.64	86.69	88.22	92.22
Formal	78	79	80	83	81	85	83.82	85.38	97.19	97.05	94.05
Shorts	89	89	90	91	89	93	91.96	93.87	92.54	92.34	92.34
Jeans	68	68	69	78	75	81	79.6	81.76	91.28	91.07	92.07
Longpants	96	96	96	95	96	96	97.18	97.74	94.68	94.56	94.56
Skirt	80	80	81	82	81	85	85.74	87.65	98.57	97.15	98.55
Facemask	72	73	76	74	73	78	76.51	79.18	88.10	89.78	93.78
logo	87	87	88	89	88	90	91.07	90.87	94.98	94.92	94.92
Stripe	55	56	55	65	60	68	70.15	68.04	88.52	88.18	95.78
mAP	80	80.5	81.3	85	82.9	86.4	85.86	87.58	91.73	92.27	93.98

### 4.3 Experimental settings

#### 4.3.1 Training

**Figure 4:**
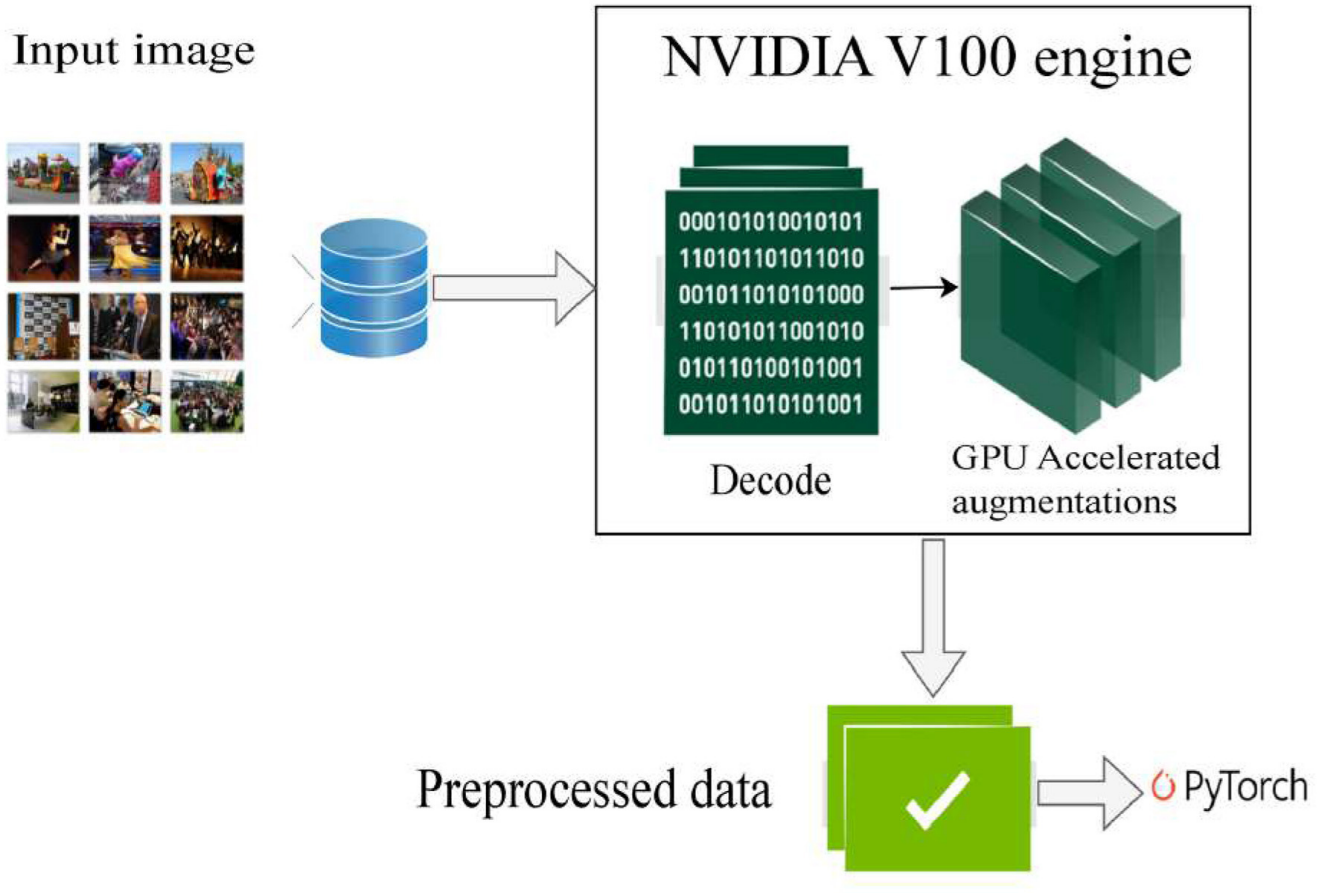
Pipeline for data processing: input MRI image processed on a GPU machine using preprocessing techniques for enhanced analysis. Reproduced with permission from ‘Recognize Complex Events From Static Images by Fusing Deep Channels' by Xiong et al via Web Image Dataset for Event Recognition [WIDER], https://paperswithcode.com/dataset/wider, licensed under CC BY-SA 4.0.

The MPAR-RCNN was trained using the Pytorch framework in an NVIDIA Tesla V100 engine. The input size of the image was 512 x 512. The augmentations used are RandomFlip, RandomMirror, RandomRotate, and RandomCrop. Hue and saturation augmentations are used to assign random colors to objects and to ensure that model will be trained to identify sharp edges instead of only colors. The images are blurred using Gaussian Distribution to reduce the effect of noise. The proposed model is trained with MogaNet as the baseline model(backbone). It was trained for 100 epochs with learning rate of 1e-4 and weight decay of 5e-5. The momentum is set to 0.9. Adam optimizer is used to minimize the loss function effectively. Experimental setup is depicted in Figure 4.

#### 4.3.2 Implementation

In MPAR-RCNN, the input image is first preprocessed by reshaping and normalized. The features extracted from the backbone architecture are passed to the RPN network detection head. This returns the label logits and box logits. Using RPN anchors, the prediction boxes are decoded and top K proposals are generated. Here, “K” is the number of objects in the image panel. The proposed BBox is forwarded to the Fast R-CNN head for accurate detection of humans or objects. After the post-processing steps, the detection head returns the final bounding boxes and the probability values with labels for objects or humans in this case. For attribute head, the features are taken from the same convolution layer as MPAR-RCNN by using an MTL pipeline, to the ROIAlign network, for the accurate localization of the attributes of the object or humans. The features are then upscaled using another convolution network and passed to the attribute head. The collective fully convolutional (FC) network is referred to as attribute head. This returns the final attributes for the given input image pixel. The proposed work is depicted in Algorithm 1.

**Algorithm 1:** Multiple person multi - person attribute recognition.

Input: Person Detection and Attributes Dataloader D, ground truth labels y, MPAR-RCNN model is initialized with weights w, total number of epochs E, Stochastic Gradient Descent Optimizer O
Output: Final Bounding Box and Attribute Predictions of the model
1: *E*←100
2: for *epoch*←0 to *E* **do**
3: for *batch*∈*D* **do**
4: ⊳ Batch contains input image I, Anchors A and ground truth tables y
5: *I, A, y*←*batch*
6: ⊳ Pass the input data to Moganet backbone to extract M_backbone to extract the *feature maps F*
7: *F*←*M*_*backbone*(*batch*)
8: ⊳ Pass the features to Box Prediction Block (BPB) *which has proposal Network R and Detection Head F*_R_*, which predicts the final boxes B and Labels L*
9: Proposals *P*←*R*(*F*)
10: *B, L*←*F*_R_(*P*)
11: ⊳ Calculate the Box Loss L_Box_
12: *L*_*Box*_←∑(*y*_*i*_−*B*_*i*_)
13: ⊳ Pass resnet C5 feature to Attribute Prediction Block(APB) which results the *predicted attribute Attr*
14: *Attr*←*APB*(*C*)
15: ⊳ Calculate the Attribute Loss L_attr_
16: $L_{attr}\left(Z\right)\leftarrow e^{z_{i}}/\sum e^{z_{i}}$
17: ⊳ Apply the Optimizer O and calculate the gradients
18: end **for**
19: end **for**

## 5 Results and discussion

The MPAR-RCNN is validated by applying distinctive frameworks and diverse functionalities, including OD, attribute recognition (AR), and MTL. The datasets used for testing the architectures with various backbones were COCO and Wider-Attribute datasets. It has bounding box detection values along with their mA values of the attributes. MPAR-RCNN is conjecturally using detection plus attributes in an MTL framework. The average precision (mAP) of the BBox, as well as the mA values of the attributes, is equally important always keeping the deal-breaker on par, i.e., the number of parameters and MACs of architectures. Table 1 depicts the comparison between the OD, AR, and the existing best metrics for MTL networks.

### 5.1 Global detection metric

**Figure 5:**
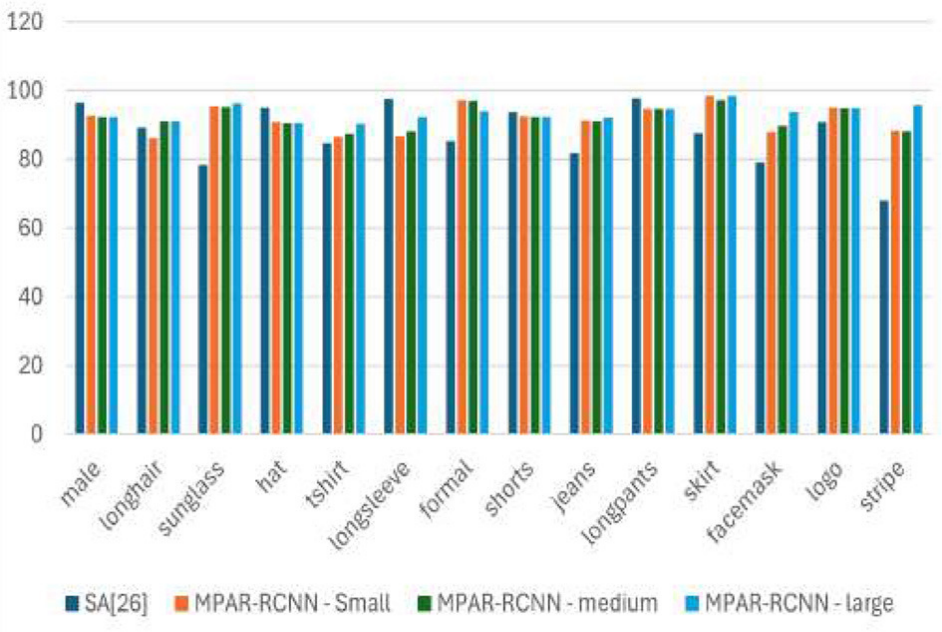
Proposed MPAR-RCNN framework shows increased mAP values for many attributes comparing with SA (Kalayeh and Shah, 2019) architecture.

**Figure 6:**
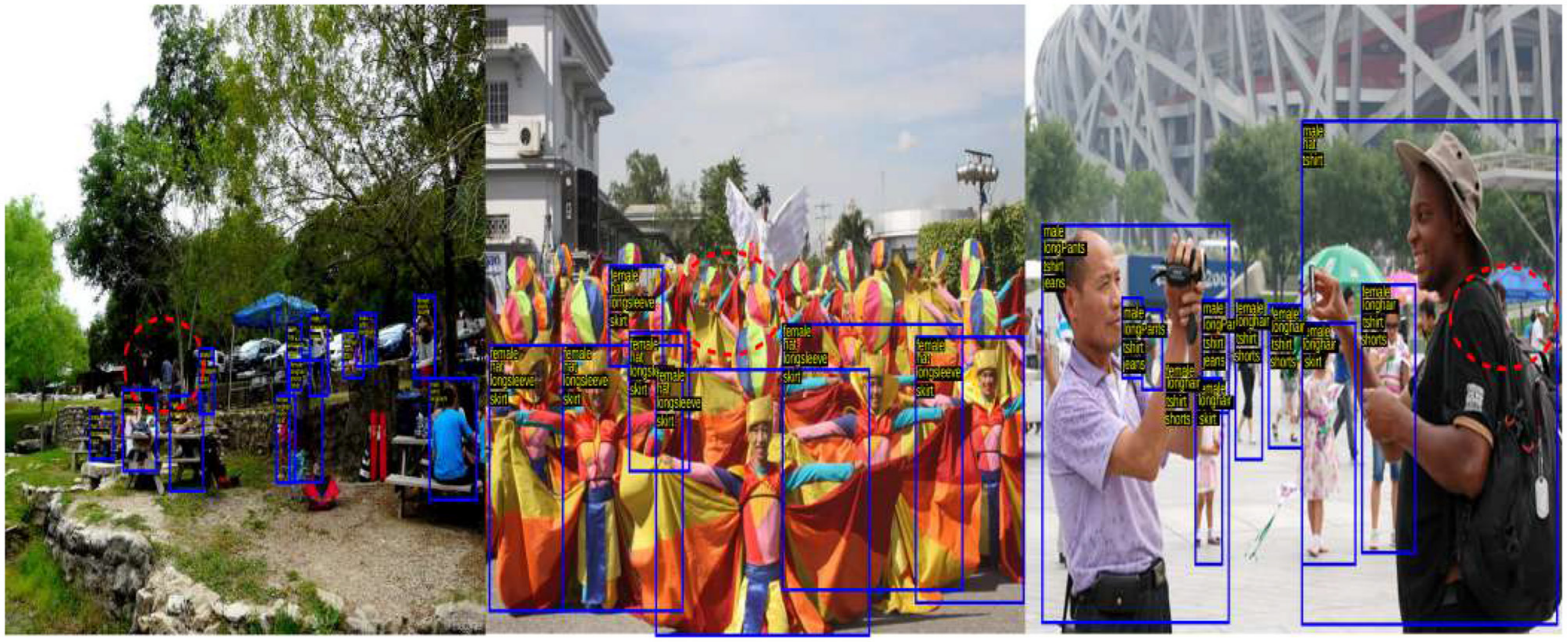
Highlighting the model's flaw in case of one object occluded by other. Reproduced with permission from ‘Recognize Complex Events From Static Images by Fusing Deep Channels' by Xiong et al via Web Image Dataset for Event Recognition [WIDER], https://paperswithcode.com/dataset/wider, licensed under CC BY-SA 4.0.

For OD, Faster-RCNN and Fast-RCNN were used to evaluate as they are the best dual-stage networks that yield robust results. R-CNN (Gkioxari et al., 2015), deep hierarchical contexts (DHC) (Li et al., 2016), VeSPA (Sarfraz et al., 2017), a refined ResNet101 (He et al., 2016), SEA (Sarafianos et al., 2018), inception V3 (Szegedy et al., 2016), and symbiotic augmentation (SA) (Kalayeh and Shah, 2019) architectures are analyzed for AR to determine their mean accuracy (mA) via the WIDER-Attribute dataset. The most recent MTL frameworks, including Mask RCNN (He et al., 2017), Parsing RCNN (Yang et al., 2019), HEIR-RCNN (Yang et al., 2020a), and RP-RCNN (Yang et al., 2020b) with two separate backbones, are compared with the proposed architecture. Different tasks were carried out for each of these MTL frameworks, for example, Mask RCNN used OD as well as object segmentation (OS). In SRN, samples from the test set were utilized to estimate training performance, and the validation set was incorporated into the training (resulting in 20% additional training data). In SEA, they re-implemented the network by training with only the training data and tested with only the test set found a difference of 1.2 in their overall mAP. This value was considered as only 20% of the testing data was reduced. Table 3 it is confident enough to argue that the proposed shared backbone MTL framework using MogaNet achieves 1.5 times better results than the previous benchmarks. The observation from Table 3 is depicted in Figure 5. There is a significant increase in mAP values for many attributes including “*s*unglass”, “*t*shirt”, “*f* ormal”, “*j*eans”, “*s*kirt”, “*f* acemask”, “*l*ogo”, “*s*tripe”. It proves that the proposed network architecture is capable to achieve more than the existing methods or at least trying to level with just tangible differences. However, the model might not accurately anticipate attribute classes when one item is obscured by another. This limitation is highlighted in Figure 6.

This section contains learning activities based on the evaluation of the testing set and the union of the training and validation sets. As far as SOTA models are compared, there is no other study that addresses both detection and attribute recognition using an MTL framework; thus comparison with definite models cannot be compared objectively. Due to this, a small modification is done to MPAR-RCNN, i.e., an in-depth analysis is done to match other research in the next section as accurately as possible. The next section gives a detailed attribute comparison of SOTA architectures using the WIDER-Attribute dataset.

### 5.2 Comparisons with state of the art

The proposed framework is evaluated with all the existing SOTA throughout the years and the latest models involving MTL networks as mentioned in the literature survey. All the architectures are evaluated on the Wider-Attribute dataset with detection and attribute recognition. It has been used as baselines, namely, Fast-RCNN (Girshick, 2015), R-CNN (Girshick et al., 2014), DHC (Li et al., 2016), VeSPA (Sarfraz et al., 2017), CAM (Guo et al., 2017), and fine-tuned ResNet101 (He et al., 2016) frameworks. Note that DHC and R-CNN have the advantage of additional spatial and temporal information (for example, scenario context or object parts) that eventually explains the boost in their performance.

#### 5.2.1 Quantitative analysis

**Figure 7:**
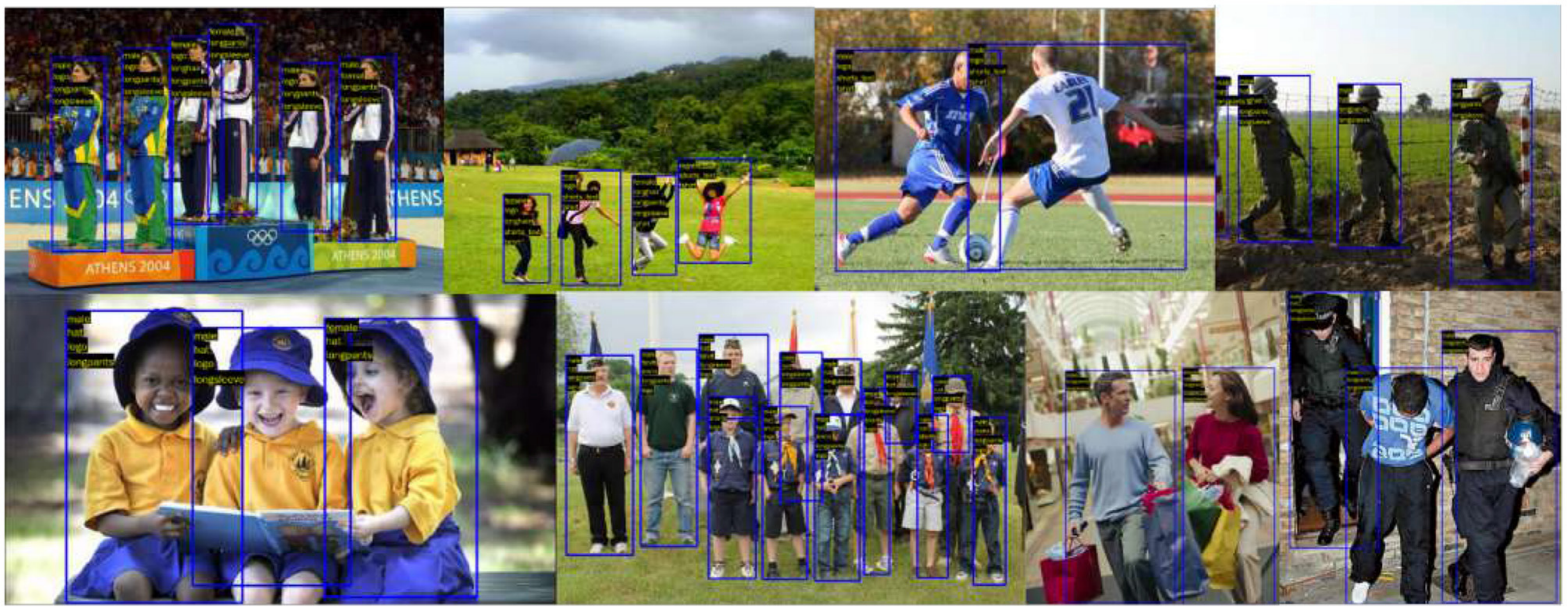
Representation of the output visualization of MPAR-RCNN framework which has the capability of detecting multiple persons with attributes. Reproduced with permission from ‘Recognize Complex Events From Static Images by Fusing Deep Channels' by Xiong et al via Web Image Dataset for Event Recognition [WIDER], https://paperswithcode.com/dataset/wider, licensed under CC BY-SA 4.0.

**Figure 8:**
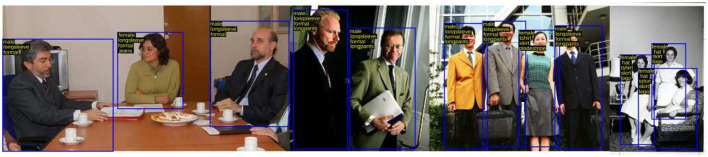
Quantitative output visualization of proposed MPAR-RCNN model. Reproduced with permission from ‘Recognize Complex Events From Static Images by Fusing Deep Channels' by Xiong et al via Web Image Dataset for Event Recognition [WIDER], https://paperswithcode.com/dataset/wider, licensed under CC BY-SA 4.0.

A fair quantitative comparison of all the existing architectures with the proposed work is displayed in Table 3. The viewpoints and attributes were parallelly predicted in the VeSPA. The model did not use the WIDER dataset to train its attribute sub-network, and hence, the attribute-wise mA values are missing from the table. Hence for overall comparison, the mAP metrics of all the SOTA architectures are used. From Table 3, the MPAR-RCNN is seen to show more than a 3% increase in overall mAP values than the existing SOTA methods. Symbiotic augmentation (SA) outputs one mask per activation channel. Therefore, it combines distinct semantic mappings to obtain the ideal mask for each channel. SA uses the attribute prediction output logits to direct the semantic segmentation, hence explaining the high performance before the MPAR-RCNN. The accuracy and the number of parameters trade-off are circumvented, after giving the top priority to the MPAR-RCNN MTL framework. The in-depth analysis of MPAR-RCNN projects that it achieves high benchmarks for attributes such as “Sunglasses”, “T-shirt”, “Formal”, “Jeans”, “Skirt”, “facemask”, “logo”, and “stripe”. The output visualization of the MPAR-RCNN Framework is shown in Figure 7. Figure 8 shows that the model can be generalized across different datasets. An eye-level view can detect up to 8–10 people in a single image. With an elevated view, approximately 7-9 people can be detected, while a pole-mounted view can also detect 7-9 people. However, it is important to note that the accuracy of attributes may vary.

## 6 Ablation study

Several ablation studies were carried out to analyze the effects of OD alone with the MPAR-RCNN with different backbones. The learning and evaluation are done on the training and validation set, respectively.

### 6.1 Attribute head analysis

**Figure 9:**
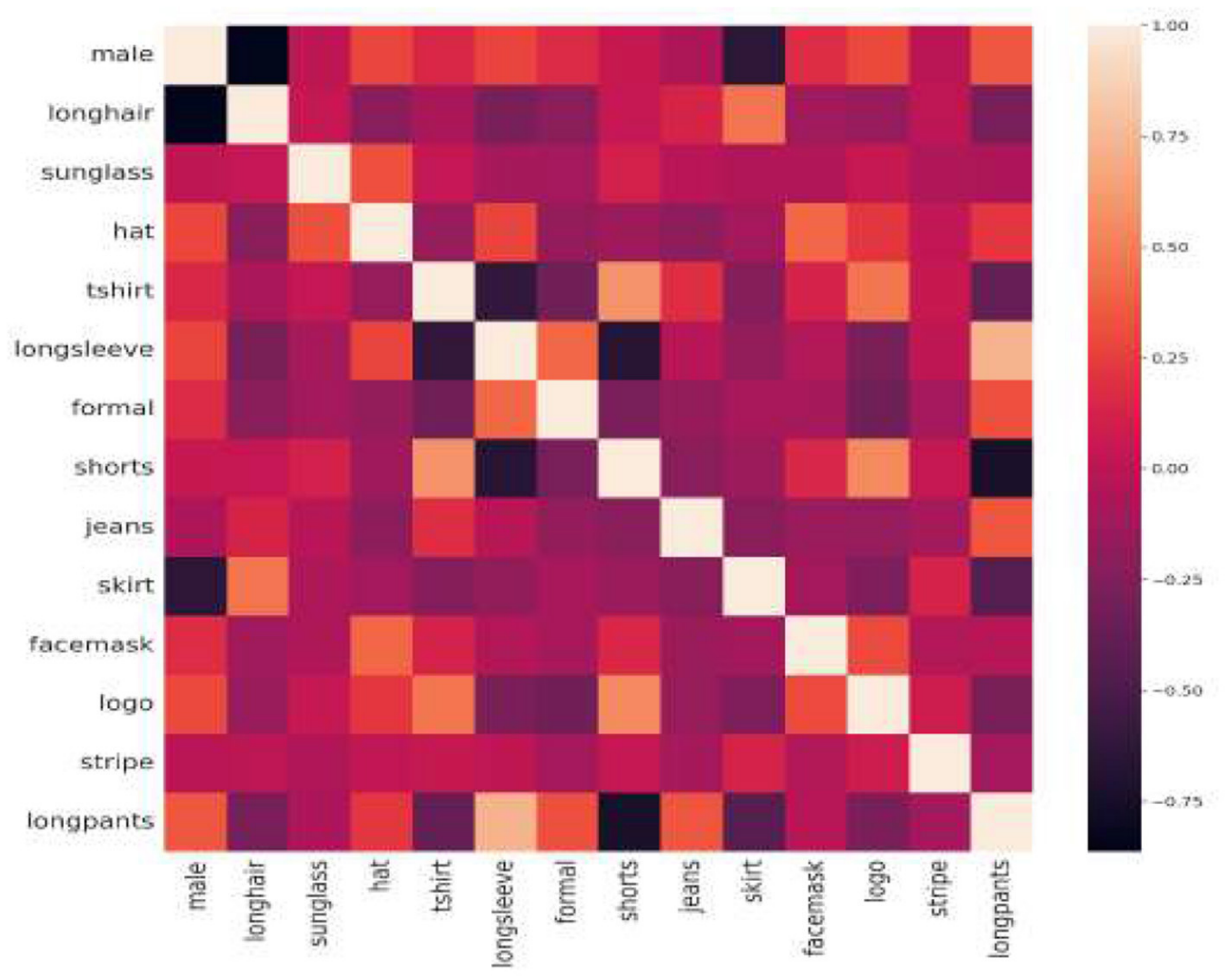
Heatmap of the correlation matrix for WIDER-Attribute dataset.

Table 4 summarizes the average precision (AP) values of gradient results ranging between 0.5 to 0.95 at their individual primes. MPAR-RCNN is analyzed with different backbone and specific number of parameters. Overall, the fork-sample version definitely produces the greatest results when compared to all other approaches. This is perhaps because just one task is taken into account per example for each backward run, eliminating much of the oversight and contributing noise. Hence, an additional experiment was conducted with different augmentations and substituting of the input size in the proposed network. The MPAR-RCNN heatmap of the correlation matrix of the WIDER-Attribute dataset is shown in Figure 9 and indicates its robust strength on each attribute. The lowest score (black in color) compares different attributes in the field. The heatmap explains the imbalance of certain attributes present in the dataset and how each attribute influences the detection with attributes in the proposed MTL framework.

**Table 4:** Comparison of proposed MPAR-RCNN network with different backnones.

**OD comparison**				
**Backbone**	**Params**	**BBox**		
		**AP(0.5:0.95)**	**AP(0.5)**	**AP(0.95)**
Resnet50	45	57.8	78.1	52.6
Swin	52	60.98	82.3	57.2
MogaNet	26	61.4	84.6	58.6

### 6.2 Enhanced prediction head

The refinement of the block is accommodated using MogaNet backbone layer and with an increased input size of 512 × 512. The bounding box AP and mAP values of different attributes of the WIDER-Attribute dataset are used. The values showcase that even with numerous attributes, the different augmentations techniques such as hue, saturation levels, and Gaussian blur help the MPAR-RCNN to achieve 93.98% accuracy level and 93.44% for only one attribute head with input size 512 × 512. These results confirm that normalizing gradients at the initial level helps in upscaling several tasks semantically. MPAR-RCNN would detect more attributes as shown in Table 5.

**Table 5:** Testing and evaluation of MPAR-RCNN variation with different augmentations and sizes.

**MPAR-RCNN with different variations and augmentations**				
**Baseline**	**Additional layer [attribute head]**	**Augmentation**	**BBox AP(0.5:0.95)**	**Attributes (mAP)**
✓		59.02	92.57
✓	✓		61.3	93.44
✓	✓	✓	61.4	93.98

## 7 Conclusion and future works

Over the years, academics and industrialists have researched more on the detection of objects. Along with detection, attribute recognition gave a more detailed description of the objects. To attain both tasks in an optimized approach with less inference time and the number of parameters and high-performance metrics. A novel multi-tasking learning framework is introduced known as MPAR-RCNN, which integrates OD plus attribute recognition with a shared backbone using a dual-stage network. With extensive experiments, MPAR-RCNN has shown that it has outperformed the existing state of the art by a marginal increase of over a percentage on the WIDER-Attribute dataset. In further ablation studies, MPAR-RCNN is benchmarked with different datasets and augmentation techniques.

Since the MPAR-RCNN is a generic and flexible framework, it can be incorporated with different backbones and other architectures. However, in some cases, the model may not be able to predict attribute classes correctly, for example, male presenter can be detected as female presenter if the person is occluded. Deploying MPAR-RCNN using transformers and graph neural networks can be considered for future research works. Adding more attributes and jointly learning for different scenarios like pedestrians with attribute recognition in ADAS based systems is also viable as the hardware in the autonomous vehicle has constraints regarding memory and size restrictions.

## Data Availability

Publicly available datasets were analyzed in this study. This data can be found here: https://paperswithcode.com/dataset/wider.
